# Diabetes mellitus in Kabuki syndrome 1 on a background of post-transplant diabetes mellitus

**DOI:** 10.1530/EDM-23-0133

**Published:** 2024-01-29

**Authors:** S Chew Sue Mei, N Pritchard, H Grayton, I Simonicova, S M Park, A I Adler

**Affiliations:** 1 Wolfson Diabetes and Endocrine Clinic, Cambridge University Hospital NHS Foundation Trust, Cambridge, UK; 2 Department of Renal Medicine, Cambridge University Hospital NHS Foundation Trust, Cambridge, UK; 3 Cambridge Genomics Laboratory, Cambridge University Hospitals NHS Foundation Trust, Cambridge, UK; 4 Department of Clinical Genetics, Cambridge University Hospitals NHS Foundation Trust, Cambridge, UK; 5 University of Oxford Diabetes Trials Unit, Oxford, UK

**Keywords:** Adolescent/young adult, Female, White, United Kingdom, Pancreas, Diabetes, Genetics and mutation, Genetics and mutation, Unique/unexpected symptoms or presentations of a disease, January, 2024

## Abstract

**Summary:**

Kabuki syndrome is a genetic disorder characterised by distinctive facial features, developmental delays, and multisystem congenital anomalies. Endocrine complications such as premature thelarche and short stature are common, whereas disorders of glycaemic control are less frequent. We describe a 23-year-old white female referred to the diabetes clinic for hyperglycaemia during haemodialysis. She was subsequently diagnosed with Kabuki syndrome based on characteristic clinical features, confirmed by detecting a heterozygous pathogenic variant in KMT2D. She was known to have had multiple congenital anomalies at birth, including complex congenital heart disease and a single dysplastic ectopic kidney, and received a cadaveric transplanted kidney at the age of 13. She had hyperglycaemia consistent with post-transplant diabetes mellitus (DM) and was started on insulin. Examination at the time revealed truncal obesity. She developed acute graft rejection and graft failure 14 months post-transplant and she was started on haemodialysis. Her blood glucose levels normalised post-graft explant, but she was hyperglycaemic again during haemodialysis at the age of 23. Given her clinical phenotype, negative diabetes antibodies and normal pancreas on ultrasound, she was assumed to have type 2 DM and achieved good glycaemic control with gliclazide.

**Learning points:**

- Involve clinical genetics early in the investigative pathway of sick neonates born with multiple congenital anomalies to establish a diagnosis to direct medical care.
- Consider the possibility of Kabuki syndrome (KS) in the differential diagnoses in any neonate with normal karyotyping or microarray analysis and with multiple congenital anomalies (especially cardiac, renal, or skeletal), dysmorphic facial features, transient neonatal hypoglycaemia and failure to thrive.
- Consider the possibility of diabetes as an endocrine complication in KS patients who are obese or who have autoimmune disorders.

## Background

Kabuki syndrome (KS) is a rare, multisystem congenital disorder described in 1981 by two groups of Japanese geneticists. Classically, people with KS have a characteristic facial appearance that resembles the makeup of traditional Japanese Kabuki theatre actors. Although first identified in Japan, KS has since been reported globally across multiple ethnicities.

The characteristic dysmorphic features are long palpebral fissures with everted lateral lower eyelids, arched laterally sparse eyebrows, a depressed nasal tip, prominent ears, missing teeth and fingertip pads, postnatal growth retardation, and intellectual disability (1, 2). The KS phenotype changes in adulthood when patients can develop truncal obesity (2). Other features of KS include cardiac anomalies (70%), renal and urinary tract anomalies (25%), skeletal defects (brachydactyly, joint hypermobility, vertebral anomalies, kyphoscoliosis), deafness (mainly conductive, but sometimes sensorineural or mixed), autoimmune diseases (vitiligo, immune thrombocytopenia, haemolytic anaemia, and diabetes mellitus (DM)), immunodeficiency with recurrent infection, as well as endocrinopathies, of which the most common are premature thelarche, obesity in adolescence, and short stature. Less common endocrinopathies include hyperinsulinism leading to transient neonatal hypoglycaemia (it is estimated that about 1% of neonates with hyperinsulinism have KS), adrenal insufficiency, combined pituitary hormone deficiency, diabetes insipidus, hypothyroidism, primary ovarian dysfunction, and true precocious puberty (1, 2). The genitourinary malformations in KS represent a broad spectrum, from undetected to severe anomalies, and include dysplasia, hydronephrosis, duplex kidney, and horseshoe kidney (3).

Following advances in molecular genetics, mutations in two causative genes have been identified in the pathogenesis of KS. Approximately 75% of patients have a pathogenic variant in the *KMT2D* (previously known as MLL2) gene inherited in an autosomal dominant manner, while 3–5% of patients have a pathogenic variant in the KDM6A gene, which is inherited in an X-linked dominant manner. Up to 30% of KS cases have no mutation found (2). Most cases of KS are *de novo* variants. Management is mainly supportive involving multiple specialties (1).

While neonatal hypoglycaemia from congenital hyperinsulinism is an established feature of KS, DM is an infrequently reported endocrine complication (4). It has been reported in obese patients and those with autoimmune DM (5). With renal transplants nowbeing more commonly performed for end-stage renal disease (ESRD), post-transplant diabetes mellitus (PTDM) is a complication. To date, there is only one post-renal transplant paediatric case of diabetes in the literature in an individual with KS treated with tacrolimus (6).

We describe a 23-year-old Caucasian female with KS1 and renal dysplasia who developed young-onset DM after a failed renal transplant in childhood and PTDM.

## Case presentation

A 23-year-old female was referred to a diabetes clinic for hyperglycaemia while on haemodialysis.

She was a preterm baby born at 35 weeks gestation via spontaneous vaginal delivery to a 34-year-old G2P1 mother and a 34-year-old father. She was the second child of nonconsanguineous, healthy parents. Complex congenital heart disease was detected on scan prior to birth; the pregnancy and labour were uncomplicated. The mother was not exposed to teratogens prenatally.

At birth, the infant was hypotonic with a low birth weight below 5 lb (near the first centile), but otherwise in reasonable condition. Her plasma creatinine was high on day 1, and an ultrasound showed a single dysplastic kidney. An echocardiogram revealed aortic coarctation, hypoplastic aortic arch, double outlet right ventricle, atrial and ventricular septal defect. She received surgery for coarctation repair and pulmonary artery banding. A karyotype revealed no abnormalities. The infant was discharged with monitoring of her renal function.

She had her second surgery at 4 months for anatomical repair of her congenital heart and pulmonary artery de-banding, and a subsequent aortic valvotomy at the age of 3. She was also diagnosed with mild global developmental delay and intellectual disability justifying special educational needs. She had significant mixed conductive and sensorineural hearing loss of both ears attributed to glue ear and aminoglycoside-induced damage, requiring bilateral hearing aids.

At the age of 13, the patient developed end-stage renal failure and was started on peritoneal dialysis. Eighteen months later, she received a cadaveric transplanted kidney from a 22-year-old male with diabetes. Her pre-transplant weight and height were 63 kg and 1.43 m, and her body mass index (BMI) was 31 kg/m^2^. Immunosuppression was maintained with tacrolimus, azathioprine and prednisolone.

During the immediate post-operative period, she had hyperglycaemia consistent with PTDM and was started on insulin. She also developed acute pulmonary oedema, complicated by a respiratory arrest, and was diagnosed with pulmonary hypertension. Two months post renal transplant, her haemoglobin A1c (HbA1c) was 7.2% (55 mmol/mol), and autoantibodies (anti-glutamic acid decarboxylase (anti-GAD) and anti-pancreatic islet-cell antibodies (anti-ICA) were negative.

Weaning her prednisolone decreased her insulin requirements. However, her renal function gradually deteriorated, and she was hospitalised 9 months post-renal transplant for acute rejection of her renal graft. She also had multiple infections, including herpes zoster, and developed acute tubular necrosis secondary to acyclovir. Her condition worsened, and the renal allograft was removed in February 2011 and she was started on haemodialysis. Her risk for another renal transplant was deemed to be too high because of her pulmonary hypertension.

Her diabetes resolved after removing the kidney graft and stopping immunosuppressive medications. Her HbA1c normalised to 5.1% (32 mmol/mol) and random capillary blood glucose levels were normal. One of her random blood glucose levels was found to be elevated 6 years later, at 16.3 mmol/L; however, all subsequent serum glucose levels were within the range of 6–9 mmol/L. At the age of 23, 9 years later, she had blood glucose values above 20 mmol/L during haemodialysis. She was referred to the diabetes clinic. Abdominal ultrasonography revealed a normal pancreas. There were no diabetic complications detected. Her family history was notable for her older brother having been diagnosed with type 1 diabetes at the age of 17.

Physical examination revealed a young woman with truncal obesity (weight 54.8 kg, height 1.43 m, consistent with a BMI of 26.8 kg/m^2^). She had coarse facial features, arched eyebrows, down-turned corners of the mouth, and a normal palate. Other findings included abnormal ears bilaterally, increased cutaneous vascularity on the left half of the chest, persistence of fetal finger pads, short fifth fingers with short fifth metacarpals bilaterally, and short fourth and fifth toes and metatarsals. The diabetologist recommended gliclazide 40 mg orally once a day and a referral to the clinical genetics team.

The geneticists suspected KS and confirmed it with molecular testing which detected a nonsense variant c.12703C>T p.(Gln4235Ter) in exon 39 of the KMT2D gene by next-generation sequencing (Illumina Dragen Bio-IT Platform, VarSeq by GoldenHelix). This was presumed to be a *de novo* mutation; her mother was deceased, and her father tested negative for the KMT2D variant.

At follow-up in the diabetes clinic 1 year later, she remained on gliclazide 40 mg once daily; her HbA1c improved from 8% (64 mmol/mol) to 6% (42 mmol/mol), and capillary blood glucose readings ranged from 4–5 mmol/L pre-breakfast to 10–12 mmol/L pre-dinner. She was subsequently found to have low serum IgG and IgA. She continued to experience limited mobility and breathlessness secondary to pulmonary hypertension.

In September 2021, the patient developed a severe coronavirus disease 2019 (COVID-19) infection. Because of her multiple complex comorbidities and rapid deterioration, the physicians and family agreed to withdraw life-sustaining measures. The patient died at 25 years of age.

## Discussion

We describe a case of non-autoimmune DM in an overweight patient with KS. Recognising the constellation of clinical features should prompt clinicians to refer to clinical genetics to consider KS among other syndromic disorders. This case study highlights the difficulties of establishing a genetic diagnosis in neonates with multiple congenital anomalies, leading to an era when genetic testing for most monogenic disorders was not available and clinical genetics was emerging as a recognised specialty (2). Whole-genome sequencing is now an early test after microarray analysis performed urgently as an agnostic test in a sick neonate suspected of having a monogenic disorder or on a routine time scale in stable children. Early molecular diagnoses help patients receive timely, targeted multidisciplinary care. They also allow families to understand the risks when choosing to expand their family and have access to appropriate reproductive options (2, 4).

Different types of diabetes have been reported in KS. T1DM was diagnosed in a Japanese girl with KS, who also was HLA-DR4 positive (5). The occurrence of T1DM in the absence of KS is usually sporadic in 90% of cases but may occurin patients with a family history of T1DM and HLA risk genotypes DR3/4-DQ8 or DR4/DR4 (6). Insulin-dependent diabetes secondary to pancreatic hypoplasia was diagnosed in another Japanese patient with the KMT2D mutation (4). In contrast, hyperinsulinaemic hypoglycaemia has been reported in cases of KS with KDM6A mutations (1). Given that our patient had a brother with type 1 diabetes, she was tested for pancreatic autoantibodies and was negative. If our patient had type 1 diabetes, then our testing did not detect it; however, the clinical course would have been unusual for type 1 diabetes.

In a case series of 21 Chinese patients in Hong Kong with KS, the risk of T2DM was reported to be 20% (4/21) in early adulthood; all patients with T2DM had the KMT2D gene and 28.6% (6/21) of these patients were obese (8). In addition, a case report described a possible association between low birth weight and an increased risk of T2DM in KMT2D KS patients, hypothesised to be because of reduced pancreatic beta cell mass and insulin resistance from obesity in late childhood (4).

Of note, an experimental study using knockout mice showed that the KMT2D histone methyltransferases were required for the induction of the peroxisome proliferator-activated receptor-γ (PPARγ) (9), which improves insulin sensitivity via multiple mechanisms including adipogenesis and glucose homeostasis. The study also found that KMT2D was required for brown adipose tissue and muscle development (9), both of which are strongly associated with insulin sensitivity. Therefore, a disruption to these processes could lead to insulin resistance and type 2 DM.

Our patient likely had PTDM and thereafter type 2 DM in view of her central obesity, raised BMI, negative T1DM antibodies, and a normal pancreas on imaging. Furthermore, she responded well to treatment with low-dose sulfonylureas. She was not tested for maternally inherited diabetes and deafness.

The experience surrounding PTDM in renal transplant patients comes mainly from adults. Some immunosuppressive agents increase insulin resistance and cause insulin deficiency (10). Corticosteroids have a dose-dependent causal association with hyperglycaemia (10). Both calcineurin inhibitors increase the risk of diabetes, which is higher with tacrolimus (14%) as compared with cyclosporine (5%) in a study of paediatric patients with liver transplants (11). Many studies address immunosuppressive protocols that incorporate early withdrawal of corticosteroids or corticosteroid-sparing regimes. However, current guidelines still recommend that the choice of immunosuppression prioritise preventing graft rejection over lowering the risk of PTDM (12).

This highlights the importance of detecting and optimising dysglycaemia pre-transplant. Current literature recommends a 2-h oral glucose tolerance test, in both paediatric (13) and adult populations (14). Fasting blood glucose may remain normal because of reduced renal clearance of insulin, and HbA1c can have limited sensitivity in ESRD patients.

## Conclusion

In conclusion, our case emphasises the importance of an early diagnosis of KS to anticipate and manage its long-term complications. Any clinician seeing a patient with DM and a history of multisystem disorders without obvious aetiology or a unifying diagnosis should consider evaluation for associated genetic syndromes. DM, renal disease, and central obesity can be manifestations of KS.

## Declaration of interest

The authors declare that there is no conflict of interest that could be perceived as prejudicing the impartiality of the case study reported.

## Funding

This work did not receive any specific grant from any funding agency in the public, commercial, or not-for-profit sectors. AIA is funded by the NIHR Oxford Biomedical Research Centrehttp://dx.doi.org/10.13039/501100013373.

## Patient consent

The patient is deceased; written informed consent was received from the patient’s father, noting she would have been very pleased to know her experience could help others.

## Author contribution statement

SC performed the literature review and wrote the manuscript. HG and IS are clinical scientists who helped run genetic tests on the patient’s samples. NP is a nephrologist, and S-MP is a clinical geneticist who reviewed and edited the manuscript. AA is a diabetologist who supervised the project.
